# Companion diagnostics: changing patient management

**DOI:** 10.3332/ecancer.2012.244

**Published:** 2012-02-22

**Authors:** Succinct Healthcare Communications

## Abstract

At the European Multidisciplinary Cancer Congress (EMCC), held in Stockholm in September 2011, a recurring theme in many of the workshops was personalised medicine, including the latest developments in prognostic and predictive biomarkers. Such markers, it is hoped, will enable clinicians to use available resources to best effects—by offering treatments to only those patients most likely to benefit, or by avoiding treatments that are likely to cause toxicities with limited benefit. The emergence of novel diagnostic tools that can distinguish subsets of patients with different response to treatment is likely to result in a paradigm shift in the way in which we manage cancer in the future. This report focuses on some of the key developments and challenges in providing a truly individualised approach to therapy, as presented at EMCC 2011.

## Introduction

In September 2011, Stockholm played host to the European Multidisciplinary Cancer Congress (EMCC), which attracted nearly 13,000 participants and 694 speakers from 116 countries. The theme for many of the Congress workshops was personalised medicine, reflecting the increasing number of research initiatives that are aiming to deliver “the right treatment to the right patients at the right time [1]”.

EMCC 2011 included 1,768 posters and 181 oral presentations, in addition to educational workshops and sponsored sessions. To summarise all of the information presented would be overwhelming. This report therefore focuses on some of the key developments and challenges in providing a truly individualised approach to therapy, providing examples of important prognostic and predictive markers that may influence future practice.

New diagnostics are making it increasingly possible to individualise, or personalise, medical therapy by identifying patients who are more likely to benefit from a particular treatment, or who are at lower or higher risk for a particular side effect. While the concept of targeted treatments is not new (table 1), the emergence of novel diagnostics that can distinguish subsets of populations that respond differently to treatments is likely to result in a paradigm shift in the way we manage patients in the future. Rational drug development allied with companion diagnostics for the identification of specific patient populations has the potential to achieve beneficial outcomes for patients, physicians, payors and industry, with shorter development cycles and fewer treatment failures than seen in the past.

## Targeted treatments and their companion diagnostics

Within the past few months, two targeted treatments have been approved by the U.S. Food and Drug Administration (FDA): vemurafenib (RO5185426, PLX4032) for the treatment of people with BRAF V600E mutation-positive metastatic malignant melanoma and crizotinib (PF-02341066) for the treatment of advanced anaplastic lymphoma kinase (ALK)-positive non-small cell lung cancer (NSCLC). In parallel, companion diagnostics have been prospectively assessed and approved by the FDA to ensure the robust and accurate selection of patients for these therapies.

### Vemurafenib for BRAF mutation-positive metastatic malignant melanoma

Vemurafenib was specifically designed to target selectively and inhibit a mutated form of the BRAF protein which is found in about half of all cases of melanoma. The phase III BRIM3 study in 675 patients with metastatic melanoma found that first-line treatment with oral vemurafenib (960 mg bid) improved overall survival compared with dacarbazine (1,000 mg/m^2^ IV every 3 weeks) with a hazard ratio (HR) of 0.44 (95% CI 0.333–0.59) after a median follow-up of 6.2 months [2]. Dose-limiting toxicity includes grade 3 fatigue, arthralgias, photosensitivity, rash, and raised alkaline phosphatase and squamous cell carcinomas (in 23% of patients treated at the maximum tolerated dose [3]).

In parallel, evaluations have shown that robust, rapid and accurate molecular testing was achieved with the companion diagnostic, Cobas^®^ 4800 BRAF V600 Mutation Test (Roche Molecular Systems Inc.) in the phase III multicentre study. Compared with 2X bi-directional Sanger sequencing, the Cobas 4800 test had a lower failure rate, was more sensitive in the detection of V600E mutations and detected most V600K mutations [4].

### ALK-positive NSCLC — patient characterisation and crizotinib treatment

In August 2011, the FDA approved crizotinib — the first tyrosine kinase inhibitor specifically targeted for the treatment of locally advanced or metastatic ALK-positive NSCLC. ALK is one of the newest tyrosine kinase targets in NSCLC, which is aberrantly activated in approximately 4% of patients [5]. Patients can be identified as ALK-positive using the companion diagnostic, Vysis ALK Break Apart FISH Probe Kit (produced by Abbott Molecular Inc [6–8]). Testing for ALK-positive NSCLC should be considered particularly in patients known to be EGFR and KRAS negative, because ALK, EGFR and KRAS mutations do not occur concurrently [5].

A series of small trials has been conducted with crizotinib. Among 119 evaluable patients, most of whom had received more than one previous line of therapy, the objective response rate was 61% and progression-free survival was 10 months following treatment with crizotinib [9]. In a subanalysis of 81, mainly young (median age 51 years), never-smokers with adenocarcinoma histology, overall survival was not reached (95% CI 17 months to not reached) after a median follow-up of 18 months [5]. Although multiple patterns of resistance to crizotinib have been described *in vitro*, data from the clinic are still lacking, reported Dr Robert Doebele from the University of Colorado Cancer Center, Aurora CO, USA.

With the first description of ALK-positive NSCLC reported in 2007, there is still much to learn about this newly-recognised patient subset. Patients with ALK-positive NSCLC appear to have a similar prognosis to the general population of patients with NSCLC [5]. However, Robert Doebele and colleagues have observed that treatment-naïve ALK-positive patients are more prone to pericardial effusions and metastatic spread to the liver than patients who have either KRAS-positive or triple-negative NSCLC (i.e. negative for ALK, EGFR and KRAS). These findings illustrate the importance of tumour biology in driving the pattern of metastatic spread.

### Draft regulatory guidance on in vitro companion diagnostics

Shortly before the approval of the companion diagnostics for vemurafenib and crizotinib, the long-awaited draft regulatory guidance for industry and FDA staff on *In Vitro* Companion Diagnostic Devices was published [10]. This user friendly guide for researchers and sponsors encourages early consultation with the FDA when developing an *in vitro* companion diagnostic device (or test).

## Challenges in the implementation of personalised therapy

Although the identification of oncogenic drivers may provide a rationale for treatment, it remains an art to integrate this information into clinical decision-making, observed by Dr Gordon Mills from the University of Texas MD Anderson Cancer Center, speaking at the Opening Session.

Today, oncologists recognise and treat more subtypes of cancer, representing more homogenous cohorts than in the past. However, currently only a small proportion of patients benefit from targeted treatments [11]. Most cancers have multiple mutational drivers, requiring multiple targeted agents or broad-targeted agents. In general, treatment responses are short-lived, and resistance mechanisms still have to be overcome in order to deliver on the promise of more personalised care.

### Passengers or drivers?

As tumours are characterised in more depth, researchers need to determine which aberrations present are likely to be “passengers” (a consequence of genomic instability or aberrations in DNA repair) and which are “drivers” (determining tumour behaviour). Dr Mills observed that, unlike patients with resectable early-stage disease, candidates for clinical trials tend to have high-grade tumours that are often driven by genomic instability and p53 mutations and only a few of these patients have controllable mutations [11]. Next-generation sequencing approaches are proposed as an important step forward in implementing personalised cancer therapy — but current techniques have only a 60% true-positive rate and a 60% false-positive rate [11], both of which Dr Mills considered unacceptable for clinical management.

Nevertheless, the University of Texas MD Anderson Cancer Center has initiated a research programme to begin to deliver on the promise of personalised medicine — identifying any aberration or mutation for which there is a targeted treatment or any mutation which occurs with a frequency of >5% on any tumour lineage [11]. Of patients referred to a phase I programme with various tumour types, PIK3CA mutations were found to be predictive of higher response to PI3K/AKT/mTOR inhibitors (35% vs. 6% in those without documented mutations); but the concomitant presence of KRAS or BRAF mutations conferred resistance in only some cancer types [12]. This early research shows that both the underlying gene expression of the tumour as well as the tumour lineage are critically important in determining the response to targeted therapy [11].

### Discordance between primary and metastatic tumours

Dr Mills said: “If we (as researchers) fail to characterise all of the subclones, we may manage only a subset of the tumour and, indeed, by eliminating the dominant clone, we could allow a more aggressive subclone to grow out” [11]. There are already data showing that patients with differences between primary tumours and metastases in terms of biomarkers fare far worse than patients without differences [11]. In breast cancer, there is discordance between the dominant tumour clones in primary and metastatic disease in approximately 40% of cases. Aberrations in PIK3CA, for example, tend to be stochastic rather than the drivers of the disease process in metastatic disease, were noted by Dr Mills.

In melanoma, positive selection for the mutated allele KIT in the metastatic tissue is associated with a prolonged response to imatinib mesylate; however, consistent selection of this mutant allele is only observed in a minority of patients [13].

## The measure of circulating tumour cells

Technologies that can detect and characterise circulating tumour cells (CTC) hold great promise as they are expected to replace metastatic tissue biopsies for the monitoring of response and resistance, as well as cancer recurrence following treatment. CTC may also be used to assess the presence or absence of certain biomarkers, which may differ from the primary tumour tissue.

Temporal changes in the number of CTCs of patients with epithelial tumours have been shown to correlate with the clinical course of disease as measured by standard radiographic methods [14]. The collection of CTCs using a microfluidic platform (such as the CellSearch^®^ system) which is capable of efficient and selective separation of viable CTCs from peripheral whole blood samples potentially provides a new and effective tool for accurate identification and measurement of the response to treatments [14,15].

The MIRACLE project (Magnetic Isolation and moleculaR Analysis of single CircuLating and disseminated tumour cElls on chip) aims to develop a fully automated and integrated microsystem providing the genotype (gene expression profile) of CTCs and disseminated tumour cells (DTCs) from bone marrow from clinical samples. The project — a collaborative venture between European universities, research institutions and companies — is funded by the European Commission. Work on the MIRACLE project started in September 2010. Further information is available at http://www.miracle-fp7.eu/

## CYP2C19*2 polymorphism predicts benefit of adjuvant tamoxifen

Genotyping for variants of the CYP2C19*2 allele found in 13% of Caucasians potentially provides another route for personalised medicine. Genotyping can help to identify patients with the CYP2C19*2 polymorphism; this is a subset of patients who derive a significantly greater survival benefit from adjuvant tamoxifen (HR 0.23; p=0.0002) than those with the CYPC19 wildtype genotype [16]. CYP2C19 is one of the enzymes which metabolises tamoxifen into its active metabolites.

Polymorphisms in tamoxifen-metabolising enzymes not only confer variation in the response to treatment but, for a subset (with the CYP2C19*2 allele), can impact on prognosis negatively if patients are not treated with adjuvant tamoxifen [16]. These results are from TAMOX trial which followed up post-menopausal women with breast cancer for a median of 9.6 years [16].

## BRCA status translates into a predictive biomarker for high-dose adjuvant chemotherapy in breast cancer

It is estimated that half of patients with breast cancer do not gain any benefit from adjuvant chemotherapy. High-dose chemotherapy has largely been abandoned in the adjuvant setting because of its toxicity and because it confers little, if any, survival benefit [17]. However, using array comparative genomic hybridization (aCGH), Dr Sabrina Linn and colleagues from The Netherlands Cancer Institute, Amsterdam, have identified a subset of patients with stage-III, human epidermal growth factor receptor 2 (HER2)-negative BRCA-associated breast cancer who achieve a significantly prolonged survival with high-dose chemotherapy (cyclophosphamide+thiotepa+car boplatin) compared with conventional therapy (fluorouracil+epirubicin+cyclophosphamide [18]). In this subset, representing approximately one-third of patients, high-dose chemotherapy achieved an 81% improvement in survival after a median follow-up of 8 years compared with conventional chemotherapy (adjusted HR 0.19, 95% CI: 0.08–0.48 [18]).

For over a decade, it has been known that breast and ovarian tumours with BRCA1 and BRCA2 germ-line mutations are hallmarked by genomic instability due to a DNA damage repair defect. Numerous sporadic tumours share these ‘BRCA-like’ traits [19]. This defect has recently been exploited as a predictive biomarker for DNA double strand break (DSB) inducing agents, such as platinum salts or bifunctional alkylators and poly (ADP-ribose) polymerase 1 (PARP-1) inhibitors [20].

## Plasma VEGFA — a potential predictive biomarker for bevacizumab

VEGF inhibitors, such as bevacizumab, have been shown to improve survival in subsets of patients [21]. Identifying reliable prognostic markers as well as predictive markers for improved response to anti-angiogenics is critical in selecting an individualised and tailored approach to this treatment. Most bevacizumab trials include pre-treatment biomarker sampling, with a focus on plasma VEGFA (pVEGFA), which has shown prognostic rather than predictive value [22]. However, recent findings using a novel ELISA-based assay for shorter isoforms (VEGFA121 and VEGFA110) suggest the potential value of these isoforms for predicting overall survival and/or progression-free survival with bevacizumab in metastatic breast (AVADO), pancreatic (AVITA) and gastric (AVAGAST) cancers, but not in metastatic colorectal (AVF2107g), non-small cell lung (AVAiL) or renal cell (AVOREN) cancers [23–25]. Prospective trials are now underway to validate these findings and to assess the potential of plasma VEGFA as a predictive biomarker in clinical practice [23].

## ImmunoPET down regulates HER2 expression in trastuzumab-refractory breast cancer

Zirconium-89 (^89^Zr), when conjugated to trastuzumab, enables positron emission tomography (PET) imaging of HER2-positive lesions. Results from a phase I study showed that the accumulation of ^89^Zr-trastuzumab in lesions allowed PET imaging of most known HER2-positive lesions as well as some that had been previously undetected in patients with metastatic breast cancer [26]. This new technology is now being used to visualise HER2 expression before and shortly after treatment. Using this immunoPET technique has provided an early insight into the *in vivo* effect of a new treatment (the heat shock protein-90 [HSP90] inhibitor, AUY922) which down regulates HER2 expression in trastuzumab-refractory breast cancer. The study (NCT01081600 [27]) is being conducted at the University Medical Centre Groningen (The Netherlands) in collaboration with the Royal Marsden Hospital (United Kingdom).

## FLT-PET — a predictive and prognostic marker in NSCLC

Since the introduction of targeted therapy into the treatment of advanced NSCLC, molecular imaging tools have gained in importance for assessing pharmacodynamics and prognosis and for predicting therapeutic outcome. In particular, 3′-deoxy-3′-[18F]fluoro-L-thymidine (FLT) uptake measured by PET is recognised as an important non-invasive marker of tumour proliferation. This marker is predictive of likely response to erlotinib [28] and has recently been found to be a strong prognostic marker for overall survival among patients with advanced NSCLC treated with erlotinib first-line [29]. PET/CT with 18F-FLT could contribute to the selection of patients that may benefit from treatment with EGFR tyrosine kinase inhibitors.

## FDG-PET provides early prediction of pathological complete response

Using functional imaging, Dr Cristina Gamez and colleagues from the University Hospital of Bellvitge, Barcelona, Spain, demonstrated the predictive value of an early [18F]-fluorodeoxyglucose [FDG]-PET response in determining those patients who would achieve a pathological complete response [30]. Of a subset of 77 women with HER2-positive breast cancer from the NEO-ALTTO study receiving neoadjuvant lapatinib and trastuzumab for 6 weeks (followed by 12 weeks of paclitaxel), those with metabolic changes in tumours identified by FDG-PET at week 2 and week 6 were twice as likely to achieve a pathological complete response at the time of surgery 12 weeks later [30].

## Conclusion

The FDA approval of the first targeted treatments and their companion diagnostics heralds a new era of precision medicine. Although a truly individualised approach to treatment is still a long way off, a wealth of research is ongoing to identify and validate predictive and prognostic biomarkers; new targets in the cell signalling pathway; novel agents that have minimal off-target activity or that overcome resistance to current therapies; new trial designs to allow enriched patient populations and rapid data collection. In the future, other innovative solutions (from the collection of CTCs) and/or novel functional imaging techniques (including immunoPET) may provide further insights into the nature of various cancers and their treatment for individuals.

## Figures and Tables

**Table 1:**
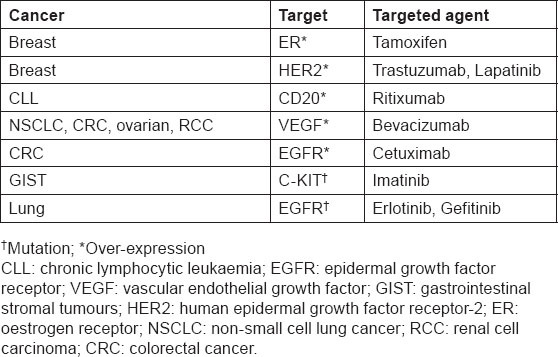
Treatments with identified targets
